# Proteomic dissection of the rice-*Fusarium fujikuroi* interaction and the correlation between the proteome and transcriptome under disease stress

**DOI:** 10.1186/s12864-019-5435-5

**Published:** 2019-01-28

**Authors:** Zhijuan Ji, Yuxiang Zeng, Yan Liang, Qian Qian, Changdeng Yang

**Affiliations:** State Key Laboratory of Rice Biology, China National Rice Research Institute, No.359 Tiyuchang Road, Hangzhou, 310006 People’s Republic of China

**Keywords:** Rice, Bakanae disease, Proteomic dissection, Transcript, Correlation

## Abstract

**Background:**

Bakanae disease, caused by the fungus *Fusarium fujikuroi*, occurs widely throughout Asia and Europe and sporadically in other rice production areas. Recent changes in climate and cropping patterns have aggravated this disease. To gain a better understanding of the molecular mechanisms of rice bakanae disease resistance, we employed a 6-plex tandem mass tag approach for relative quantitative proteomic comparison of infected and uninfected rice seedlings 7 days post-inoculation with two genotypes: the resistant genotype 93–11 and the susceptible genotype Nipponbare.

**Results:**

In total, 123 (77.2% up-regulated, 22.8% down-regulated) and 91 (94.5% up-regulated, 5.5% down-regulated) differentially expressed proteins (DEPs) accumulated in 93–11 and Nipponbare, respectively. Only 11 DEPs were both shared by the two genotypes. Clustering results showed that the protein regulation trends for the two genotypes were highly contrasting, which suggested obviously different interaction mechanisms of the host and the pathogen between 93 and 11 and Nipponbare. Further analysis showed that a noticeable aquaporin, PIP2–2, was sharply upregulated with a fold change (FC) of 109.2 in 93–11, which might be related to pathogen defense and the execution of bakanae disease resistance. Certain antifungal proteins were regulated in both 93–11 and Nipponbare with moderate FCs. These proteins might participate in protecting the cellular integrity required for basic growth of the susceptible genotype. Correlation analysis between the transcriptome and proteome revealed that Pearson correlation coefficients of R = 0.677 (*P* = 0.0005) and R = − 0.097 (*P* = 0.702) were obtained for 93–11 and Nipponbare, respectively. Our findings raised an intriguing result that a significant positive correlation only in the resistant genotype, while no correlation was found in the susceptible genotype. The differences in codon usage was hypothesized for the cause of the result.

**Conclusions:**

Quantitative proteomic analysis of the rice genotypes 93-11and Nipponbare after *F. fujikuroi* infection revealed that the aquaporin protein PIP2–2 might execute bakanae disease resistance. The difference in the correlation between the transcriptome and proteome might be due to the differences in codon usage between 93-11and Nipponbare. Overall, the protein regulation trends observed under bakanae disease stress are highly contrasting, and the molecular mechanisms of disease defense are obviously different between 93 and 11 and Nipponbare. In summary, these findings deepen our understanding of the functions of proteins induced by bakanae disease and the mechanisms of rice bakanae disease resistance.

**Electronic supplementary material:**

The online version of this article (10.1186/s12864-019-5435-5) contains supplementary material, which is available to authorized users.

## Background

Rice (*Oryza sativa* L.) is one of the world’s most important food crops, serving as a staple for more than 50% of the world population. In China, rice is a staple food crop that feeds more than 60% of the population and contributes nearly 40% of the total calorie intake [1]. Under diverse ecological conditions, rice is frequently exposed to a variety of biotic and abiotic stresses [2]. Among biotic stresses, many diseases play an important role in determining the yield and cost of food. Rice bakanae disease, caused by the fungus *Fusarium fujikuroi*, is an important disease in rice. That can be soilborne or seedborne and results in characteristic symptoms of excessively elongated seedlings with chlorotic stems and leaves. Bakanae disease occurs widely throughout Asia and Europe, and sporadically in other areas of rice production [3, 4]. Recent changes in climate and in cropping patterns have aggravated the disease. Although bakanae disease can be managed to a certain extent using chemical fungicides [5] through seed treatment and soil amendment, a more efficient, sustainable and environmentally friendly solution is to explore genetic resources that are resistant to the disease and breed additional high-resistance genotypes.

Several studies have made progress in identifying quantitative trait loci (QTLs) governing resistance against bakanae in rice [2, 6, 7]. Yang et al. [6] first obtained two QTLs, *qB1* and *qB10*, associated with more than 13% of expressed variance for resistance to bakanae disease, via artificial inoculation at the seedling stage using a *japonica*/*indica* double haploid (DH) population derived from Chunjiang 06 and TN1. Hur et al. [7] reported a major QTL for bakanae disease resistance, *qBK1*, which explained 65% of the total phenotypic variation in near-isogenic lines (NILs) obtained from a cross between the resistant donor YR24982–9-1 and the susceptible parent Ilpum. Fiyaz et al. [2] identified three QTLs (*qBK1.1*, *qBK1.2* and *qBK1.3*, which accounted for 4.76, 24.74 and 6.49% of phenotypic variation, respectively) using a recombinant inbred line (RIL) population derived from the *indica* rice parents Pusa 1342 and Pusa Basmati 1121. *qBK1.1* is likely the same as *qBK1* identified by Hur et al. [7], but the associated phenotypic variations were extremely different because different mapping populations were used; thus, these QTLs are far from application in marker-assisted introgression of bakanae resistance in rice breeding. Recent research [8] using a GWAS approach to screen a *japonica* rice germplasm collection revealed two new genomic regions that were highly associated with the observed phenotypic variation for the response to bakanae infection on the short arm of chromosome 1 (designated qBK1_628091) and the long arm of chromosome 4 (designated qBK4_31750955), representing new genomic regions associated with *F. fujikuroi* resistance.

In addition, RNA sequencing (RNA-seq) has been used to illustrate the molecular mechanism of the interaction between *F. fujikuroi* and rice plants. Different expression patterns were identified for the resistant genotype 93–11 and the susceptible genotype Nipponbare through comparative transcriptome analysis [9]. The results revealed that certain WRKYs, WAK and MAP3Ks were responsible for the bakanae disease resistance of 93-11and showed that the defense-related genes (WRKYs and MARKs) on chromosome 1 that are modulated in 93–11 upon infection might play a crucial role in the rice-*F. fujikuroi* interaction. Matić S et al. [10] found that PR1, germin-like proteins, glycoside hydrolases, MAP kinases, and WRKY transcriptional factors were up-regulated in the resistant genotype Selenio upon infection with *F. fujikuroi*, whereas up-regulation of chitinases and down-regulation of MAP kinases and WRKY transcription factors were found in the susceptible cultivar Dorell. Pathways including the response to chitin, JA-dependent signalling and the hypersensitive response were found to be involved in bakanae resistance.

In addition to QTL mapping and transcriptome analysis of rice bakanae disease resistance, other technologies and methods are needed to further dissect resistance to bakanae disease. With the completion of rice genome sequencing in 2002, proteomics is becoming an increasingly powerful tool for the investigation of complex cellular processes and has also been successfully used for genetic and physiological studies [11]. Proteomic technology is also playing a key role in developing an inventory of proteins that are responsive to biotic and abiotic stresses and in dissecting rice defense pathways [12]. In rice breeding, proteomics is usually applied to detect stress-responsive proteins through comparisons among stressed plants. Further identification of candidate proteins may reveal that some of these proteins have functions that are clearly consistent with the stress-tolerance trait [13].

A comparative proteomics study of rice genotypes that are resistant and susceptible to bakanae disease has yet not to be reported, nor has the correlation between the proteome and transcriptome under disease stress. In this study, the tandem mass tag (TMT) technique was applied to compare the differences in the proteomic pattern associated with the bakanae disease response using the resistant genotype 93–11 and the susceptible genotype Nipponbare. On the basis of our previous study of the disease response at different transcriptome levels between the two genotypes [9], correlation analysis was conducted between proteomic data and the transcriptomic data. The molecular mechanism of rice resistance to bakanae disease was thereby comprehensively dissected.

## Results and discussion

### Responses of 93–11 and Nipponbare after inoculation with F. Fujikuroi

**Fig. 1:**
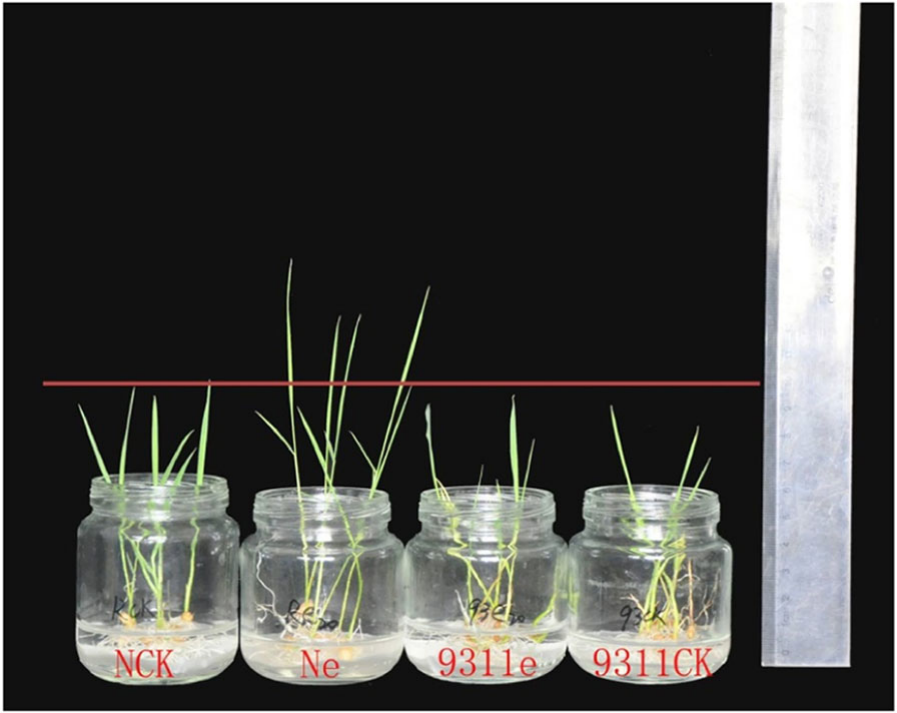
Symptoms of Nipponbare and 93–11 under inoculation with 8.8 × 10^6^ spores/ml of *F. fujikuroi*

Using the inoculation method applied in our previous study [9], 93–11 and Nipponbare were found to show different symptoms following inoculation with 8.8 × 10^6^ spores/ml of *F. fujikuroi*. Nipponbare Seedlings were elongated while 93–11 showed no elongation (Fig. 1). The seedling height of Ne and Nck presented an extremely significant difference, whereas no difference was found between 93-11e and 93-11ck (Table 1).

**Table 1:** Height of elongated seedling of 93–11 and Nipponbare treated with 8.8 × 10^6^ spores/ml of *F. fujikuroi*

Treatments	Seedling height
93-11ck	6.63 ± 0.40
93-11e	7.13 ± 0.23
Nipponbare-ck	7.70 ± 0.36
Nipponbare-e	10.03 ± 0.42**

**represents a significant difference at *P* = 0.01

Previous reports have revealed that *indica* exhibits higher resistance to rice blast (*Magnaporthe grisea*) and sheath blight (*Rhizoctonia solani*) than *japonica* [14–16]. Hur et al. [7] used the resistant *indica* variety Shingwang and the susceptible *japonica* variety Ilpum to generateNILs for identifying QTLs associated with bakanae disease resistance. In other studies, both resistant and susceptible *indica* genotypes [2] or *japonica* genotypes [8, 17] have been used to evaluate resistance to bakanae disease. In this study, Nipponbare (*japonica*) was more sensitive to *F. fujikuroi*, whereas 93–11 (*indica*) was resistant, indicating that *indica* 93–11 exhibited higher resistance to the bakanae disease stress than *japonica* Nipponbare cultivar*.*

### Protein identification and quantification

After TMT and LC-MS/MS analysis of *F. fujikuroi*-infected seedlings and mock-infected seedlings collected at 7 days post-inoculation (dpi), a total of approximately 100,000 polypeptides were detected with 48,195 and 52,576 polypeptide species being identified for 93–11 and Nipponbare respectively (Table 2). After two-stage mass spectrometry and protein quantification, the identified proteins were screened in the NCBI database (https://www.ncbi.nlm.nih.gov/, file: txid39947-Oryza-sativa-28,555 s-20150412.fasta) with VennPlex software. Overall, 7, 478 and 9161 proteins were identified in the two genotypes when a false discovery rate (FDR) < 1% was applied to the dataset.

**Table 2:** Results of protein identification and quantification

Item	93–11	Nipponbare
Polypeptides identified	100,507	106,997
Polypeptide species identified	48,195	52,576
Quantitative proteins	7478	9161
DEPs	123	91
Up-regulated DEPs	95	86
Down-regulated DEPs	28	5

**Fig. 2:**
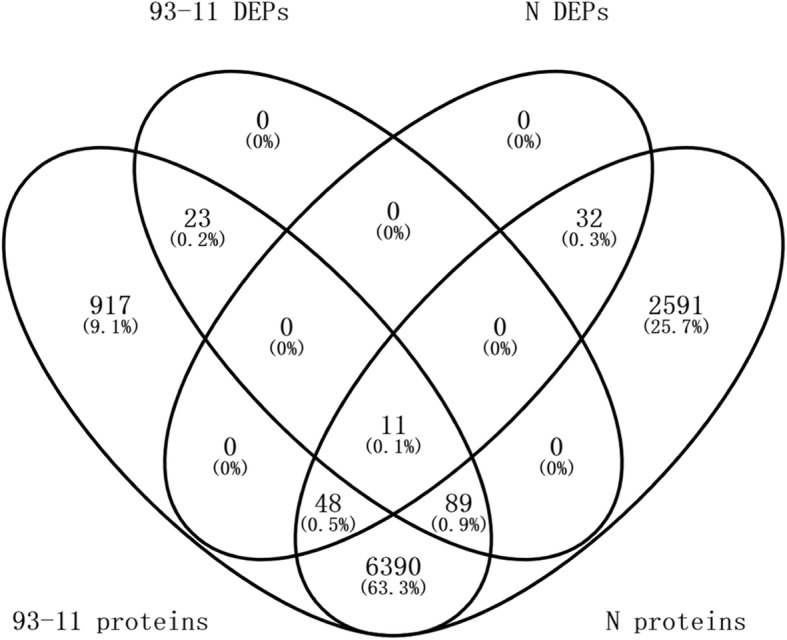
Venn diagram of total and *F. fujikuroi*-modulated proteins

A total of 123 and 91 DEPs accumulated in 93–11 and Nipponbare respectively. Using the criteria of *P* value ≤0.05 and FC ≥ 1.5 or FC ≤ 0.667, 95 (77.2%) and 28 (22.8%) DEPs in 93–11, 86 (94.5%) and 5 (5.5%) DEPs in Nipponbare were found to be up- and down-regulated, respectively. Furthermore, 11 DEPs were both shared by the two genotypes (Fig. 2).

**Fig. 3:**
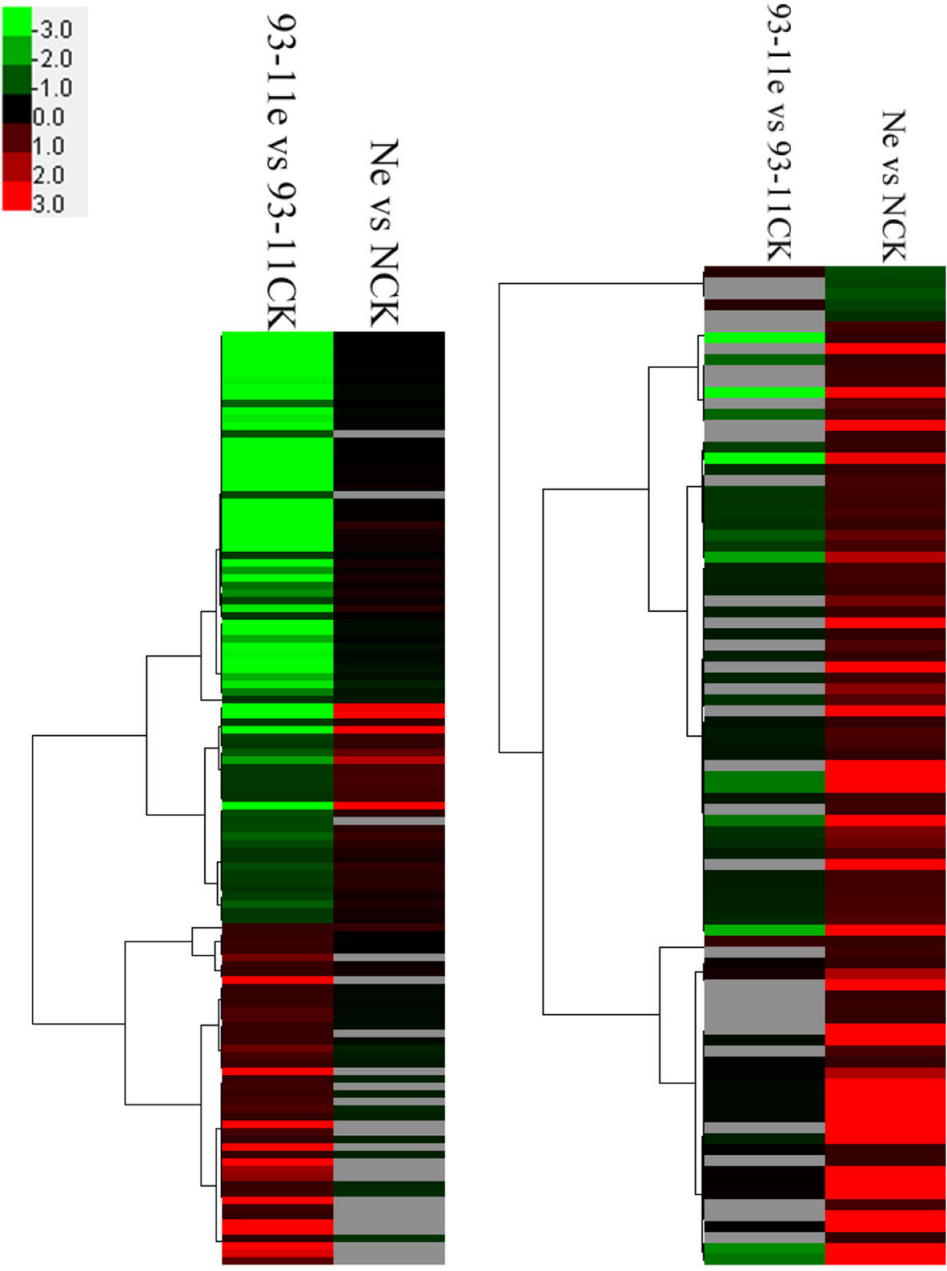
Hierarchical cluster analysis of protein expression based on FC data

Since only 11 DEPs were shared by the two genotypes, the difference in DEPs between 93 and 11 and Nipponbare infected by the pathogen were further investigated using cluster analysis (Fig. 3). Clustering results showed that the protein regulation trends for the two genotypes were highly contrasting, which suggested an obviously different interaction mechanism of the host and the pathogen between 93 and 11 and Nipponbare.

### Greatly up-regulated proteins might be closely related to bakanae disease resistance

To obtain target proteins to analyze the interaction mechanisms of the host and the pathogen, further criteria (≥2 unique peptides and ≥ 6 counts) were used for the DEPs in the two genotypes. With these further criteria, 29 (Table 3) and 27 (Table 4) DEPs were obtained in 93–11 and Nipponbare respectively, with 4 DEPs (Table 5) being shared. It was noted that the aquaporin (AQP) PIP2–2 was sharply up-relgulated with an FC of 109.2 in 93–11 (Table 3).

**Table 3:** The 29 DEPs identified in 93–11 using the further criteria

Accession	Gene_Symbol	Description	FC
XP_015626399.1	LOC4330049	probable aquaporin PIP2–2	109.18
XP_015614532.1	LOC4348376	vacuolar-sorting receptor 3	16.01
XP_015639310.1	LOC4339191	probable sugar phosphate/phosphate translocator At4g32390	7.86
XP_015622682.1	LOC4328618	fumarylacetoacetase	7.46
XP_015646863.1	LOC4344152	serine carboxypeptidase II-3	7.23
XP_015622762.1	LOC4329858	isocitrate dehydrogenase [NAD] regulatory subunit 1, mitochondrial isoform X2	3.82
XP_015618186.1	LOC4351745	glucuronoxylan 4-O-methyltransferase 1	3.48
XP_015634447.1	LOC4335518	alpha-humulene synthase	2.08
XP_015635507.1	LOC9271676	phenylalanine ammonia-lyase-like	1.91
XP_015638718.1	LOC4337901	fasciclin-like arabinogalactan protein 11	1.88
XP_015649427.1	LOC4345604	alpha carbonic anhydrase 7	1.80
XP_015622103.1	LOC4326471	salt stress-induced protein	1.80
XP_015626729.1	LOC4330040	phenylalanine ammonia-lyase	1.67
XP_015621390.1	LOC4325129	peroxidase 72	1.63
XP_015641088.1	LOC4340665	probable peroxygenase 4	1.63
XP_015623983.1	LOC4329036	salt stress root protein RS1-like	1.62
XP_015628637.1	LOC4332648	uncharacterized protein LOC4332648	1.62
XP_015651336.1	LOC4347224	1-aminocyclopropane-1-carboxylate oxidase 1	1.58
XP_015621495.1	LOC4324284	salt stress root protein RS1	1.56
XP_015641648.1	LOC4340788	UDP-glycosyltransferase 708A6	1.55
XP_015621166.1	LOC4325960	scopoletin glucosyltransferase	1.53
XP_015639141.1	LOC4339222	peroxidase 1	1.53
XP_015612645.1	LOC4347709	ribonuclease 1	1.51
XP_015623954.1	LOC4328586	peroxiredoxin-2E-2, chloroplastic	0.65
XP_015619408.1	LOC4352014	ribulose bisphosphate carboxylase small chain A, chloroplastic	0.64
XP_015627384.1	LOC4329821	putative UDP-rhamnose:rhamnosyltransferase 1	0.63
XP_015625272.1	LOC4326593	50S ribosomal protein L31	0.62
XP_015614152.1	LOC4348979	protein TIC110, chloroplastic	0.61
XP_015617238.1	LOC4326583	carbonic anhydrase, chloroplastic isoform X1	0.56

**Table 4:** The 27 DEPs identified in Nipponbare using the further criteria

Accession	Gene_Symbol	Description	FC
XP_015633534.1	LOC4335790	beta-fructofuranosidase, insoluble isoenzyme 2	11.58
XP_015617184.1	LOC4349693	G-rich sequence factor 1 isoform X1	10.98
XP_015622762.1	LOC4329858	isocitrate dehydrogenase [NAD] regulatory subunit 1, mitochondrial isoform X2	4.50
XP_015624063.1	LOC4330051	defensin Tm-AMP-D1.2	4.22
XP_015644849.1	LOC9271516	uncharacterized protein LOC9271516	3.07
XP_015649880.1	LOC4344615	cysteine-rich repeat secretory protein 55	2.70
XP_015644657.1	LOC9270927	pathogenesis-related protein 1	2.54
XP_015634447.1	LOC4335518	alpha-humulene synthase	2.22
XP_015643864.1	LOC4341249	peroxidase P7	1.86
XP_015620386.1	LOC4352160	alpha-dioxygenase 1 isoform X1	1.79
XP_015651336.1	LOC4347224	1-aminocyclopropane-1-carboxylate oxidase 1	1.78
XP_015636635.1	LOC4334987	subtilisin-like protease SBT3.8 isoform X2	1.77
XP_015621390.1	LOC4325129	peroxidase 72	1.75
XP_015631105.1	LOC4332568	pathogenesis-related protein 1	1.70
XP_015614030.1	LOC4348474	uncharacterized protein LOC4348474	1.65
XP_015651086.1	LOC4325834	uncharacterized protein LOC4325834	1.63
XP_015649764.1	LOC4345934	tricin synthase 1	1.61
XP_015620489.1	LOC4352487	major allergen Dau c 1	1.60
XP_015615445.1	LOC4350299	cytochrome P450 87A3-like	1.57
XP_015629807.1	LOC4325264	GDSL esterase/lipase At5g45910	1.56
XP_015618576.1	LOC4351585	actin-7	1.55
XP_015625928.1	LOC4330873	1-aminocyclopropane-1-carboxylate oxidase 1-like isoform X2	1.55
XP_015649937.1	LOC4346299	monodehydroascorbate reductase	1.53
XP_015641256.1	LOC4340800	cytochrome P450 76C2	1.51
XP_015632670.1	LOC4325859	salicylic acid-binding protein 2	1.50
XP_015633866.1	LOC4337415	protochlorophyllide reductase A, chloroplastic	0.57
XP_015620637.1	LOC4352505	lipoxygenase 2.1, chloroplastic isoform X1	0.51

**Table 5:** The 4 DEPs shared by the two genotypes according to the further criteria

Accession	Gene_Symbol	Description	FC for N	FC for 93–11
XP_015622762.1	LOC4329858	isocitrate dehydrogenase [NAD] regulatory subunit 1, mitochondrial isoform X2	4.50	3.82
XP_015634447.1	LOC4335518	alpha-humulene synthase	2.22	2.08
XP_015651336.1	LOC4347224	1-aminocyclopropane-1-carboxylate oxidase 1	1.78	1.58
XP_015621390.1	LOC4325129	peroxidase 72	1.75	1.63

AQP belongs to a highly conserved group of membrane proteins referred to as major intrinsic proteins that facilitate water transport across biological membranes in all types of organisms. It has been reported that AQP responds to various stresses [18], including the overexpression of *TaAQP8* in tobacco to increase tolerance to salt stress [19], overexpression of *RWC3* in rice to increase tolerance to drought stress [20], and overexpression of BnPIP1 in tobacco to increase tolerance to water stress [21]. Zhang et al. [22] suggested that aquaporin proteins might improve plant abiotic resistance through alleviating water deficit or oxidative damage. In *Arabidopsis*, AQP was shown to be involved in responses to both abiotic and biotic stresses. For instance, the aquaporin PIP2–5 is up-regulated in the roots and aerial parts of *Arabidopsis* by both drought and cold stresses [23]. Overexpression of AtPIP1;4 enhances water flow and facilitates germination in response to cold stress [24]. Regarding biotic stress resistance, it was demonstrated that the aquaporin TaPIP1 transgenic *Arabidopsis* enhanced resistance to *Pseudomonas syringae* pv. *Tomato* (*Pst*) DC3000 infection [18]. In our study, the aquaporin protein PIP2–2 exhibited 109.18-fold up-regulation after *F. fujikuroi* inoculation, which is thought to be related to pathogen defense and the execution of bakanae disease resistance. Therefore, the present study provides the second report of the role of the aquaporin protein in biotic stress resistance.

Plant vacuoles are vital organelles [25]. Perturbation of the vacuolar trafficking machinery affects many cellular processes, including responses to pathogens [26]. Wang et al. [27] found that vacuolar trafficking mediated by vacuolar sorting receptor1 (VSR1) is required for osmotic stress-responsive ABA biosynthesis and osmotic stress tolerance. In this study, a vacuolar-sorting receptor 3(VSR3) was significantly up-regulated, with an FC of 16.01 in 93–11, which meant that the vacuolar-sorting protein VSR3 could improve resistance to bakanae to some extent.

According to the the susceptible genotype Nipponbare, the FCs of the genes encoding the aquaporin and VSR3 proteins were around 1 (data in the Additional file 1), which meant that no obvious change was demonstrated for the proteins between the treatment and the control plants in the susceptible genotype.

Plants have developed various mechanisms, including the production of antifungal proteins or resistance-related proteins to defend themselves against pathogenic fungi. In this study, antifungal proteins such as defensin, peroxidase and ribonuclease were regulated in both 93–11 and Nipponbare, although the FCs of these proteins were only moderately high. Furthermore, although Nipponbare was susceptible to bakanae disease, the corresponding gene encoding the beta-fructofuranosidase protein LOC4335790, also known as CIN2/GIF1, was up-regulated with an FC of 11.58. According to functional annotation with the Uniprot database (http://www.uniprot.org/uniprot/Q0JDC5), sugar homeostasis mediated by CIN2/GIF1 plays an important role in constitutive and induced physical and chemical defense against pathogens. Yuan et al. [28] reported that genes involved in plant defenses were up-regulated when senescence was induced in a susceptible rice variety after attack by the brown planthopper and that these genes might participate in protecting the cellular integrity required for the progression and completion of senescence. Therefore, antifungal or defense-related proteins in the susceptible genotype Nipponbare might participate in protecting the cellular integrity required for basic growth of the cultivar.

### Correlation analysis between the proteome and transcriptome

A single protein-profiling experiment can provide only a few clues regarding the function of a protein [13]. The integration of available transcriptomic data with proteomic data is critical to add further value to the single proteomic datasets and to provide a global picture of gene regulation and metabolic networks. To better clarify potential molecular mechanisms, some studies have been performed to correlate protein and mRNA expression levels in the same sample, in an attempt to identify reliable genes for crop breeding [29–31].

In this study, correlation analysis was conducted at the differential expression level by combining the proteomic results of this study and the transcriptomic results of our previous study [9]. Correlation was considered to occur if a gene that was modulated as a DEP was also expressed as a DEG (differentially expressed gene at the transcript level) after *F. fujikuroi* treatment.

**Fig. 4:**
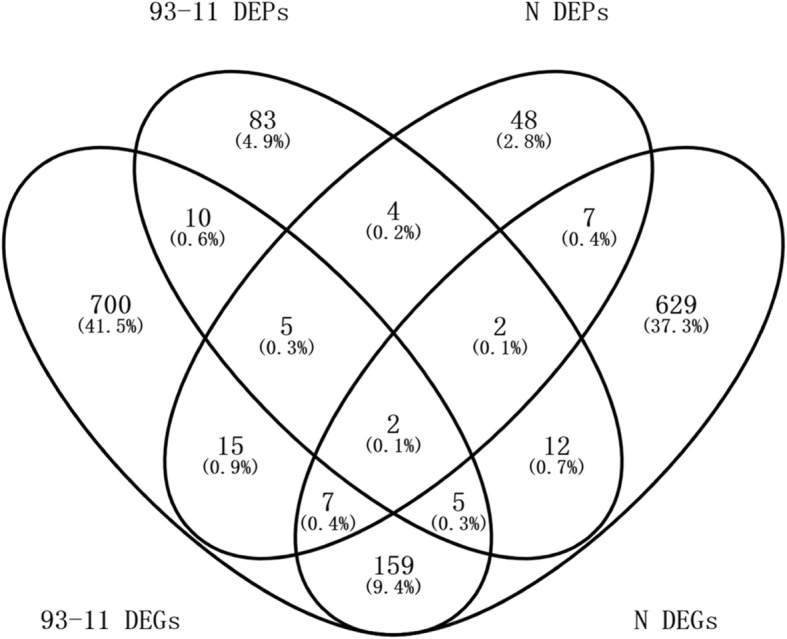
Venn diagram of DEPs and DEGs for Nipponbare and 93–11

A total of 22 DEPs (17.9% of the total DEPs) were correlated with DEGs (2.1% of the total DEGs) in 93–11, and 18 DEPs (19.8% of the total DEPs) were correlated with DEGs (1.8% of the total DEGs) in Nipponbare (Fig. 4).

The concordance between the changes in abundance at the transcript and protein levels was investigated using the corrgram function of the R programming language. Pearson correlation coefficients of R = 0.677 (*P* = 0.0005) and R = − 0.097 (*P* = 0.702) were obtained for 93–11 and Nipponbare, respectively, which meant that, a significant positive correlation was demonstrated in the resistant genotype at the two omics levels, whereas no such correlation was found in the susceptible genotype. Olivares-Hernández et al. [32] reported that most of the variability in the protein-transcript relationship can be explained by the variation in the codon composition, and codon usage variability therefore determines the correlation between proteome and transcriptome FCs. We hypothesize that the difference in the correlation of the two omic levels between the resistant and the susceptible genotypes might be due to the differences in the codon composition between the two genotypes under disease stress, resulting in the differences in the translation mechanism between the two genotypes. Further research is requisite to dissect the intriguing result.

Some arguments regarding this aspect have been put forth. In several reports, only a weak correlation was found between mRNA and protein abundances, due to post-transcriptional and post-translation regulation and measurement errors [33–35]. Ji et al. [31] obtained a value of *R* = 0.56 between the transcriptome and proteome. Certain opposite change trends were demonstrated between the two omics levels. The regulation of the process leading from mRNA to protein is generally very complex. During protein translation from mRNA, factors including mRNA splicing, altered protein turnover, protein degradation, or a combination of the above may lead to a poor correlation between the expression patterns of mRNAs and their respective proteins [36]. Komatsu [13] maintained that transcript and protein levels cannot be correlated, due to the inability of total mRNA to be translated into protein. The identification of proteomes that are not fully complete due to current limitations of proteomics [37] also affects global comparative studies of transcript and protein expression. Maier et al. [35] considered the correlation between mRNA and protein abundance to depend on various biological and technical factors. In general, with future advances in identification and quantification techniques employed in omics analyses, a more accurate picture of the organism-wide correlation between mRNA and protein will be achieved.

## Conclusions

Protein expression plays an important role in the response to environmental stresses. In this study, TMT-based experiments were performed to identify proteins that were differentially accumulated between *F. fujikuroi*-infected and mock-inoculated seedlings of the bakanae disease-resistant and -susceptible genotypes 93-11and Nipponbare, respectively, to determine the key proteins involved in disease defense. The results revealed that the greatly up-regulated protein PIP2–2 might be related to pathogen defense and the execution of bakanae disease resistance. The vacuolar-sorting protein VSR3 can improve resistance to bakanae to some extent. Certain antifungal proteins were regulated in both the resistant and susceptible genotypes. Correlation analysis of transcriptome and proteome levels revealed a significant positive correlation only in the resistant genotype, while no such correlation was found in the susceptible genotype, which could be explained by the differences in codon usage. Overall, the molecular regulation of rice bakanae disease resistance was found to be completely different between 93-11and Nipponbare. Functional verification of the key protein PIP2–2 will be a prerequisite of our future research, which may be accomplished through transferring the related gene to the susceptible genotype or knocking down the gene in the resistant genotype.

## Methods

### Plant materials and inoculation with *F. Fujikuroi*

The *indica* rice genotype 93–11 and the *japonica* genotype Nipponbare (sourced from Qian Qian’s laboratory at China National Rice Research Institute) were inoculated with *F. fujikuroi*. 93–11 is resistant and Nipponbare is susceptible to bakanae disease. The details of the inoculation method were the same as in our previous study [9]. For inoculum production, an isolate of *F. fujikuroi* xj1105 isolated from Chinese bakanae-infected rice plants was used. The fungus was grown for 7 days on potato dextrose agar (PDA) solid medium at 28 °C in the dark, after which microconidia were harvested using sterile distilled water and the concentration was adjusted to 8.8 × 10^6^ spores/ml. Rice seeds were surface sterilized in 70% ethanol for 1 min, then in 5% sodium hypochlorite for 5 min and subsequently were rinsed 5 times in sterile water. Surface sterilized seeds were germinated for three days to uniform size and vigor and were then subjected to *F. fujikuroi* stress treatment. Germinated seeds were soaked in the spore suspension and shaken for 24 h at 30 °C, while the control seeds for each genotype were soaked in sterile distilled water. The germinated seeds were further transferred to wet test paper to grow to seedlings at 28–30 °C, with a 12 h photoperiod. At the time point of 7 dpi, the seedling length of the treated plants (designated 93-11e and Ne) and the corresponding controls (designated 93-11ck and Nck) was calculated. The seedlings were further frozen in liquid nitrogen immediately after collection and stored at − 80 °C. Three biological replicates were performed.

### Protein extraction

Samples were ground in liquid nitrogen with a mortar and pestle. The sample powder was then dissolved (vortex blending) with trichloroacetic acid (TCA)-acetone and left to stand for at least 12 h at − 20 °C in precooling tubes. The mixture was then centrifuged in a Allegra 64R rotor (BeckMAN, USA) at 14,000 g at 4 °C for 15 min to remove the supernatant, and the precipitate was suspended in acetone at − 20 °C for 2 h. The suspension was centrifuged at 14,000 g at 4 °C for 10 min to remove the supernatant, and the precipitate was washed with chilled acetone and 90% acetone before vacuum drying. The lyophilized precipitate was cracked into smaller molecules with a protein breaker (diagenode, Belgium) at 4 °C for 1 h using protein extraction buffer (8 M urea, 0.1% SDS) containing additional 1 mM phenylmethylsulfonyl fluoride and a protease inhibitor cocktail (Roche, USA), and followed by centrifugation at 14,000 g for 10 min. The supernatant was carefully collected, and the protein quality was tested with a bicinchoninic acid (BCA) protein assay kit. Sodium dodecyl sulfate polyacrylamide gel electrophoresis (SDS-PAGE) was also carried out to test protein quality with a 12% cross-linked polyacrylamide gel. Protein bands on the gel were visualized with silver staining. The gels was scanned with a Molecular Imager (Bio-Rad Laboratories, USA).The protein samples were stored at − 80 °C until further processing.

### TMT labeling

The tandem mass tags TMT^6^ (Pierce, USA) with different reporter ions (126–131 Da) were applied as isobaric tags for relative quantification, and TMT labeling was performed according to the manufacturer’s instructions. Briefly, 100 μg per condition was transferred to a new tube, and 100 mM triethyl ammonium bicarbonate (TEAB) buffer was added to the protein solution to a final volume of 100 μL. Then, 5 μL of 200 mM Tris (2-carboxyethyl) phosphine (TCEP) was added, and the sample was incubated at 55 °C for 1 h. Next, 5 μL of 375 mM iodoacetamide was added to the sample, followed by incubation for 30 min at room temperature while protected from light. The proteins were precipitated with pre-chilled (− 20 °C) acetone. After resuspension with 100 μL of 100 mM TEAB, the proteins were digested overnight at 37 °C with 2.5 μg trypsin (Sigma, USA). The digested samples were individually labeled at room temperature for 1 h as follows: TMT^6^ reagents were used for 93–11, where the controls, with three replicates, were labeled with TMT^6^–126, 127 and 128, and the treatments were labeled with TMT^6^–129, 130 and 131; TMT^10^ reagents were used for Nipponbare, where the controls, with three replicates, were labeled with TMT^10^–126, 127C, and 127 N, the treatments were labeled with TMT^10^-128C, 128 N and 129 N, respectively. The labeling reaction was quenched by adding 8 μL of 5% hydroxylamine. Finally, the labeled peptide aliquots were combined for subsequent fractionation.

### Fractionation of labeled peptides

For the fractionation of labeled peptides, samples were first lyophilized and reconstituted in solvent A (2% ACN, pH 10). Then, the samples were loaded onto an Xbridge PST C18 Column (130 Å, 5 μm particle size, 250 × 4.6 mm; Waters, USA) and resolved via a basic RPLC method using a gradient of 5 to 95% solvent B (90% ACN, pH 10) in 40 min. A total of 40 fractions were collected, which were then concatenated into 20 fractions, vacuum dried and stored at − 80 °C until further LC-MS/MS analysis.

### LC-MS/MS analysis

LC-MS/MS analysis was performed at CapitalBio Technology using a Q Exactive mass spectrometer (Thermo Scientific, CA). The peptide mixture was separated through reversed-phase chromatography in a DIONEX nano-UPLC system using an Acclaim C18 PepMap100 nano-Trap column (75 μm × 2 cm, 2 μm particle size) (Thermo Scientific, USA) connected to an Acclaim PepMap RSLC C18 analytical column (75 μm × 25 cm, 2 μm particle size) (Thermo Scientific, USA). Before loading, the sample was dissolved in sample buffer containing 4% acetonitrile and 0.1% formic acid. A linear gradient from 3 to 30% mobile phase B (0.1% formic acid in 99.9% acetonitrile) over 48 min followed by a steep increase to 80% mobile phase B in 1 min was used at a flow rate of 300 nL/min. The nano-LC was coupled online to the Q Exactive mass spectrometer using a stainless steel emitter coupled to a nanospray ion source. Mass spectrometry analysis was performed in a data-dependent manner with full scans (350–1600 m/z), which were acquired using an Orbitrap mass analyzer at a mass resolution of 70,000 at 400 m/z in Q Exactive. The twenty most intense precursor ions from a survey scan were selected for MS/MS from each duty cycle and detected at a mass resolution of 35,000 at an m/z of 400 in an Orbitrap analyzer. All the tandem mass spectra were produced by the higher-energy collision dissociation (HCD) method. Dynamic exclusion was set at 18 s.

### Data analysis and TMT quantification

Proteome Discoverer software (version 1.4) (Thermo Scientific, USA) was used for database searching against the NCBI *Oryza sativa Japonica Group* database. The following criteria were applied: precursor mass tolerance of 10 ppm and fragment mass tolerance of 20 mmu. Trypsin was specified as the digesting enzyme, and 2 missed cleavages were allowed. Cysteine carbamidomethylation and TMT modifications (N-terminus and lysine residues) were defined as fixed modifications and methionine oxidation and phosphorylation as variable modifications. The results were filtered using the following settings: only high-confidence peptides with a global FDR < 1% based on a target-decoy approach were included in the results. In the TMT quantitation workflow, the most confident centroid method was used with an integration window of 20 ppm. For protein quantitation, only unique peptides were employed. Proteins with a *P* value ≤0.05 and FC ≥ 1.5 or ≤ 0.5 between the treatment and the control plants were recognized as significant DEPs.

### Protein hierarchical cluster analysis

Cluster analysis was performed for all of the DEPs identified in the 93-11ck vs. 93-11e and Nck vs. Ne comparisons on May 5th, 2018. The FCs for each DEP were clustered with Cluster 3.0 software (http://bioservices.capitalbio.com/xzzq/rj/3885.shtml) using the hierarchical method, and the results were visualized using Treeview software [38].

### Transcriptome study and correlation analysis the proteome and transcriptome

Transcriptome study was conducted in our previous report [9] via RNA-Seq and bioinformatics. Correlation was considered to occur if a gene that was modulated as a DEP was also expressed as a DEG after *F. fujikuroi* treatment. Pearson correlation coefficients were calculated using the R programming language (R version 2.15.3) to evaluate the concordance of the changes in abundance at the transcript and protein levels.

## Additional file

Additional file 1: The total proteins and DEPs of 93-11 and Nipponbare. (XLSX 9906 kb)

## Abbreviations

AQP: Aquaporin
DEGs: Differentially expressed genes at the transcript level
DEPs: Differentially expressed proteins
dpi: days post-inoculation
FC: Fold change
FDR: False discovery rate
PDA: Potato dextrose agar
QTLs: Quantitative trait loci
RIL: Recombinant inbred line
RNA-seq: RNA sequencing
TMT: Tandem mass tag
